# Annexin A5 derived from matrix vesicles protects against osteoporotic bone loss via mineralization

**DOI:** 10.1038/s41413-023-00290-9

**Published:** 2023-11-09

**Authors:** Guanyue Su, Demao Zhang, Tiantian Li, Tong Pei, Jie Yang, Shasha Tu, Sijun Liu, Jie Ren, Yaojia Zhang, Mengmeng Duan, Xinrui Yang, Yang Shen, Chenchen Zhou, Jing Xie, Xiaoheng Liu

**Affiliations:** 1 Institute of Biomedical Engineering, West China School of Basic Medical Sciences & Forensic Medicine, Sichuan University, Chengdu, 610041 China; 2 State Key Laboratory of Oral Diseases, National Clinical Research Center for Oral Diseases, West China Hospital of Stomatology, Sichuan University, Chengdu, 610041 China

**Keywords:** Osteoporosis, Osteoporosis

## Abstract

Matrix vesicles (MVs) have shown strong effects in diseases such as vascular ectopic calcification and pathological calcified osteoarthritis and in wound repair of the skeletal system due to their membranous vesicle characteristics and abundant calcium and phosphorus content. However, the role of MVs in the progression of osteoporosis is poorly understood. Here, we report that annexin A5, an important component of the matrix vesicle membrane, plays a vital role in bone matrix homeostasis in the deterioration of osteoporosis. We first identified annexin A5 from adherent MVs but not dissociative MVs of osteoblasts and found that it could be sharply decreased in the bone matrix during the occurrence of osteoporosis based on ovariectomized mice. We then confirmed its potential in mediating the mineralization of the precursor osteoblast lineage via its initial binding with collagen type I to achieve MV adhesion and the subsequent activation of cellular autophagy. Finally, we proved its protective role in resisting bone loss by applying it to osteoporotic mice. Taken together, these data revealed the importance of annexin A5, originating from adherent MVs of osteoblasts, in bone matrix remodeling of osteoporosis and provided a new strategy for the treatment and intervention of bone loss.

## Introduction

Osteoporosis is a systemic skeletal disease characterized by low bone mineral density and deteriorated bone microarchitecture, which result in an increase in bone fragility and susceptibility to fracture.^1,2^ Due to aging and reduced estrogen levels, bone loss generally occurs in elderly individuals, especially postmenopausal women, seriously threatening their health. The robust skeleton undergoes well-coordinated osteoblast-mediated bone formation and osteoclast-mediated bone resorption under physiological conditions. However, decreased activity of osteoblasts and attenuated bone formation may disrupt this balance, thus resulting in excessive bone loss and even osteoporosis.^3^ Osteoblasts are the primary cell type responsible for bone mineralization by synthesizing new collagenous organic matrix and releasing matrix vesicles (MVs).^4,5^ Notably, MVs are widely recognized as the essential event to initiate and determine mineral formation, which is responsible for the accumulation of calcium and phosphate, as well as deposition of hydroxyapatite in the collagenous matrix.^6,7^

MVs are small extracellular vesicles (EVs) budding from the plasma membrane of calcifying cells, with a size range of 30–300 nm in diameter and enrichment of DNAs, RNAs, lipids, and proteins.^8,9^ Since MVs were first detected in 1967 by Anderson^10^ and Bonucci,^11^ the role of MVs in the mineralization of bone, cartilage, and dentin has gradually been proven by increasing in vivo and in vitro studies.^12–14^ In contrast to other extracellular vesicles, MVs are enriched in tissue nonspecific alkaline phosphatase (TNAP), phosphatidylserine, and annexins (Anx), which can specifically bind to the extracellular matrix (ECM) with high affinity and serve as the initial sites of hydroxyapatite (HA) formation.^15,16^ Apart from initiating mineralization by interacting with the ECM, MVs can also communicate with other cells, such as bone marrow mesenchymal stem cells (BMSCs) and smooth muscle cells (SMCs), as transporters to induce their osteogenic differentiation under either physiological or pathological conditions.^17–19^ Although the importance of MVs in ECM mineralization and bone formation is widely acknowledged, the underlying mechanisms of MV-mediated osteogenic differentiation and ECM interactions remain poorly understood.

Reports focused on membrane biology show that a large class of proteins known as annexins, which are located on the outer membrane of MVs, play a fundamental role in the functions of MVs.^20–22^ Annexin A, a calcium ion-dependent phospholipid binding protein, is widely expressed in various tissues throughout the body and is involved in a variety of biological processes, such as intracellular signal transduction, vesicle transport, and the formation of mineral phases in the extracellular matrix.^23,24^ Annexin A subfamily members, including AnxA1, AnxA2, AnxA4, AnxA5, AnxA6, AnxA7 and AnxA11, have been shown to participate in the mineralization process with abundant expression in MVs.^25,26^ However, previous studies have primarily focused on annexin-sensitive calcium influx in MV-mediated mineralization^27^ while ignoring other biological functions, such as aggregation,^28^ adhesion,^29^ and signal transduction.^30^ As a result, a better understanding of the molecular mechanisms underlying annexin-mediated MV-induced mineralization, particularly in osteoporosis, could help to elucidate the characteristics and functions of MVs and gain a new understanding of diseases that require the participation of mineralization.

In the current study, given the potential role of annexins derived from MVs in biomineralization, we hypothesized that decreased annexins in MVs might lead to an impaired mineralization capacity of osteoblasts, thus leading to osteoporotic bone loss. To determine whether and how annexins derived from MVs mediated mineralization, we first isolated MVs from both normal and osteoporotic osteoblasts and examined the expression changes of annexins. We then identified the role of MV-derived AnxA5 in mineralization and explored its underlying biomechanism in vitro and in vivo. Through this systematic investigation, we aimed to determine the changes in MVs in osteoporotic progression and demonstrate the importance of AnxA5 in MV-mediated mineralization, thus providing new clues for potential targets for bone loss in osteoporosis from the perspective of MVs.

## Results

### Adherent osteoblast-derived matrix vesicles show mineralization potential

**Fig. 1:**
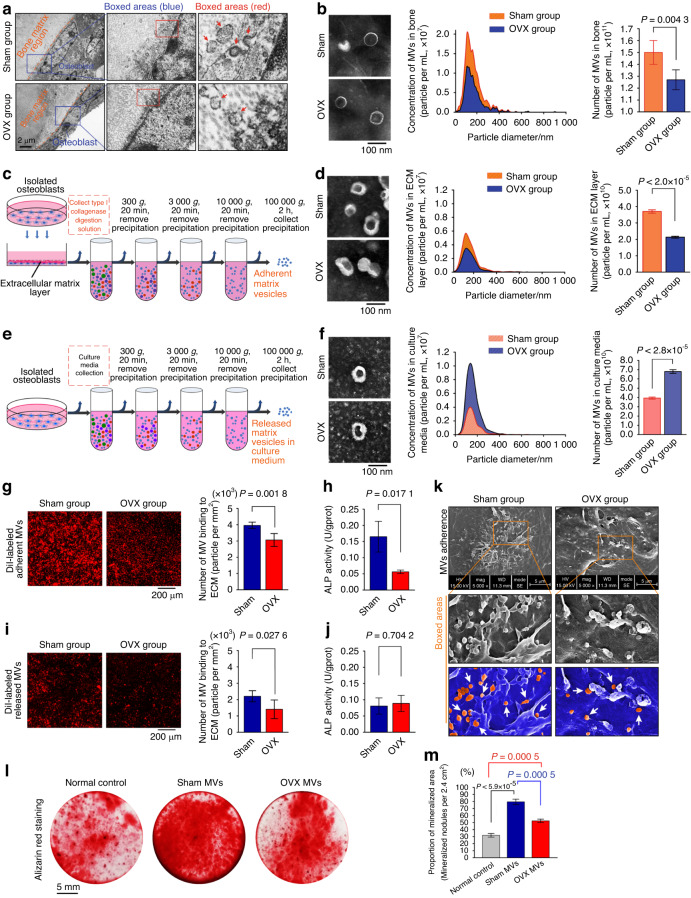
Adherent osteoblast-derived MVs show mineralization potential. **a** TEM images showing MVs secreted by osteoblasts in the bone of distal femurs. **b** TEM (left) and NTA (right) showing the morphology and number changes of MVs isolated from the cortical bone of normal and osteoporotic mice (*n* = 5). **c** Schematic diagram showing the isolation of adherent MVs from the extracellular matrix layer. **d** TEM (left) and NTA (right) showing the morphology and number changes of adherent MVs secreted by primary osteoblasts isolated from normal (Sham) and osteoporotic (OVX) bone (*n* = 3). **e** Schematic diagram showing the isolation of released MVs from culture media. **f** TEM (left) and NTA (right) showing the morphology and number changes of released MVs secreted by primary osteoblasts isolated from normal and osteoporotic bone (*n* = 3). **g** The adhesive capacity of adherent MVs secreted by normal and osteoporotic osteoblasts after 72 h co-incubation with decellularized ECM (*n* = 5). **h** ALP activity of adherent MVs from normal and osteoporotic osteoblasts (*n* = 3). **i** The adhesive capacity of released MVs secreted by normal and osteoporotic osteoblasts after 72 h co-incubation with decellularized ECM (*n* = 5). **j** ALP activity of released MVs from normal and osteoporotic osteoblasts (*n* = 3). **k** SEM images showing the changes in adherent MVs attached to the decellularized ECM substrates of osteoblasts in each group. **l** Alizarin red staining showing the formation of mineralized nodules in BMSCs treated with normal or osteoporotic MVs for 7 days. **m** Quantitative analysis indicating the changes of mineralized area (*n* = 3). Data in (**b**, **d**, **f**, **g**, **h**, **i**, **j** and **m**) are presented as the mean ± SD. The significant data in (**b**, **d**, **f**, **g**, **h**, **i** and **j**) were based on two-tailed Student’s *t* tests, while (**m**) was based on one-way analysis of variance (ANOVA) with Tukey’s post hoc tests

Matrix vesicles are key participants involved in the mineralization of extracellular matrix and bone formation.^13^ To identify the origin of and changes in MVs in the development of osteoporosis, we first constructed an osteoporotic mouse model by using bilateral ovariectomy as previously reported.^31,32^ The bone of distal femurs in normal (sham) or osteoporotic (OVX) mice was then dissected and observed by scanning electron microscopy (SEM) (Fig. 1a). We found that MVs were located in the bone matrix surrounding osteoblasts and could be secreted by budding from the plasma membrane of osteoblasts. The MVs are secreted into the extracellular space, followed by deposition in the bone matrix or transportation to other locations by blood circulation.^18^ Accordingly, MVs in cortical bone and serum of normal and osteoporotic mice were isolated to further explore the characteristic changes in MVs. Transmission electron microscopy (TEM) and nanoparticle tracking analysis (NTA) showed that MVs exhibited a bilayer cup-like morphology, with a diverse range of sizes of 30–300 nm in diameter in cortical bone (Fig. 1b) and serum (Fig. S1a), which are consistent with their typical characteristics.^33,34^ Of note, we found that the number of MVs from the bone of osteoporotic mice was significantly reduced in cortical bone (Fig. 1b, right) but the MVs showed a remarkable accumulation in serum (Fig. S1, right) compared to those in normal mice. To further characterize the MVs from osteoblasts of normal and osteoporotic tissues, we collected MVs of osteoblasts from the extracellular matrix layer (Fig. 1c) and culture media (Fig. 1e). By using TEM and NTA, we found that adherent MVs from the extracellular matrix layer of osteoblasts in osteoporosis were significantly decreased (Fig. 1d), while released MVs into culture media of osteoblasts in osteoporosis were increased (Fig. 1f) relative to those in the normal groups.

We next characterized these adherent and released MVs from normal and osteoporotic osteoblasts. The MVs labeled with DiI were seeded onto the decellularized ECM substrates, and the changes in adhesive capacity were detected (Fig. 1g-adherent MVs and Fig. 1i-released MVs). The results showed that the number of osteoporotic MVs binding to the ECM was significantly decreased compared with that of normal MVs for both adherent (Fig. 1g) and released MVs (Fig. 1i). We then examined the changes in ALP activities of MVs. Notably, adherent MVs from osteoblasts in osteoporosis showed weaker ALP activity than those in the sham group (Fig. 1h), while released MVs from osteoblasts in osteoporosis showed no difference in ALP activity relative to those of the sham group (Fig. 1j), suggesting that adherent MVs might exhibit a stronger mineralization capacity. Therefore, we focused on adherent MVs (the following MVs represent adherent MVs unless otherwise stated). To further investigate the effect of MVs on ECM adhesive capacity, we assessed the attachment of MVs onto the decellularized ECM substrates by SEM (Fig. 1k). The results indicated that MVs from osteoblasts in the osteoporotic group exhibited a visible decrease in adhesive capacity compared with those of the sham group. To investigate the effect of MVs on the mineralization capacity of BMSCs, we performed alizarin red staining (Fig. 1l). After 7 days of osteogenic induction, we found that MVs accelerated the formation of mineralized nodules in BMSCs, even MVs from osteoporotic osteoblasts. The quantitative analysis further confirmed this result (Fig. 1m). Overall, we characterized adherent and released MVs from normal and osteoporotic osteoblasts and found the importance of adherent MVs in mineralization.

### AnxA5 significantly decreased in matrix vesicles of osteoporotic osteoblasts

**Fig. 2:**
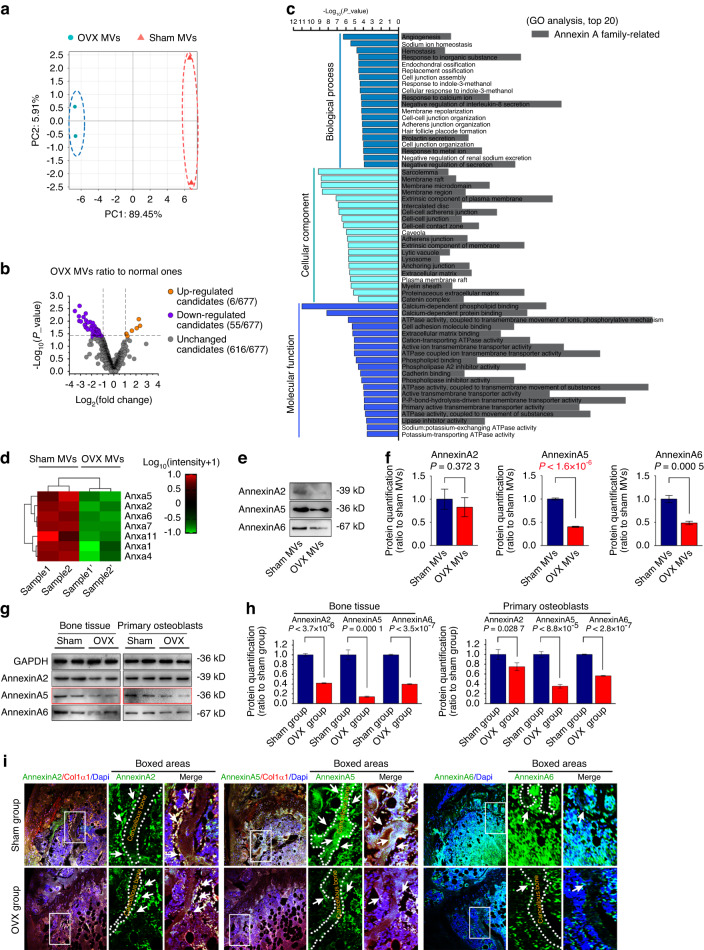
AnxA5 expression was significantly decreased in MVs in osteoporotic mice. **a** Principal component analysis (PCA) showing the correlation between the MVs from normal and osteoporotic osteoblasts (*n* = 2). **b** Volcano plot based on proteomics illustrating the differentially expressed proteins in MVs from normal and osteoporotic osteoblasts (*n* = 2). **c** GO analysis showing the top 20 biological processes, cellular components and molecular functions in normal and osteoporotic MVs. **d** Pheatmap demonstrating the protein changes of annexin A family in normal and osteoporotic MVs (*n* = 2). **e** Western blot showing the expressions of AnxA2, AnxA5 and AnxA6 in normal and osteoporotic MVs. **f** Protein quantification of AnxA2, AnxA5 and AnxA6 in each group (*n* = 3). **g** Western blot showing the expressions of AnxA2, AnxA5 and AnxA6 in long bone tissues and primary osteoblasts of normal and osteoporotic mice. **h** Quantification of AnxA2, AnxA5 and AnxA6 in each group (*n* = 3). **i** Immunofluorescence by CLSM showing the expression of AnxA2, AnxA5 and AnxA6 in normal and osteoporotic cancellous bones (green: AnxA2/AnxA5/AnxA6, red: Col1α1, blue: Nucleus). White dashed lines indicate the expression of AnxA2, AnxA5 and AnxA6 at the sites of osteoblast distribution. Data in (**f**, **h**) are presented as the mean ± SD, and Significant differences were based on two-tailed Student’s *t* tests

The function of MVs is closely related to their contents. Thus, we examined the changes in the protein profiles of normal and osteoporotic MVs by using protein mass spectrometry (Fig. 2a–c). After confirmation of sample validity through PCA (Fig. 2a), we found all differentially expressed proteins (DEPs) of MVs from osteoblasts in the normal and osteoporotic groups and presented them in a volcano plot (Fig. 2b). We identified 61 DEPs: 6 upregulated and 55 downregulated proteins. Among them, annexin family members have attracted increased attention due to their potential effects on matrix mineralization. We then performed GO analysis on these DEPs and found that annexin A was closely related to a variety of cellular components, the most important of which was membrane structure. Moreover, we found that annexin A is involved in various molecular functions, such as calcium-dependent phospholipid/protein binding, cell adhesion binding, and ECM binding, and participates in mineralization-related biological processes, including response to inorganic substances and response to calcium ions (Fig. 2c).

To further explore the role of annexin A in MV-mediated mineralization, we clustered all changed members of the annexin A family in a pheatmap (Fig. 2d). The results showed that there were seven downregulated members in osteoporotic MVs, of which annexin A5 (AnxA5), annexin A2 (AnxA2) and annexin A6 (AnxA6) showed the most obvious decrease. We then confirmed the protein expression of AnxA2, AnxA5 and AnxA6 by Western blotting and found that AnxA5 showed a higher basal expression in MVs than the other two, and its loss in osteoporotic MVs was greater than that of AnxA6, while the protein level of AnxA2 showed no significant change (Fig. 2e, f). In addition, we detected the expression of AnxA2, AnxA5 and AnxA6 in bone tissues in vivo and in primary osteoblasts in vitro from sham and osteoporotic mice by Western blotting (Fig. 2g, h). The results showed that compared to those of the sham group, all three proteins were remarkably downregulated in both bone tissues and isolated osteoblasts of osteoporotic mice, and AnxA5 showed the greatest downregulation. Immunofluorescence staining of AnxA2, AnxA5 and AnxA6 was then performed in bone tissues to further indicate their distribution (Fig. 2i). We found that the expression of AnxA2, AnxA5 and AnxA6 was distributed along the edge of cancellous bone where the osteoblasts were distributed, and their expression was lower in the osteoporosis group than in the sham group. Importantly, the expression change of AnxA5 was shown to be the most obvious (Fig. S2). Taken together, these findings established a positive relationship between osteoporosis and loss of annexins, particularly AnxA5, which may be crucial for MV-mediated biomineralization.

### AnxA5 mediates the mineralization of the precursor osteoblast lineage

**Fig. 3:**
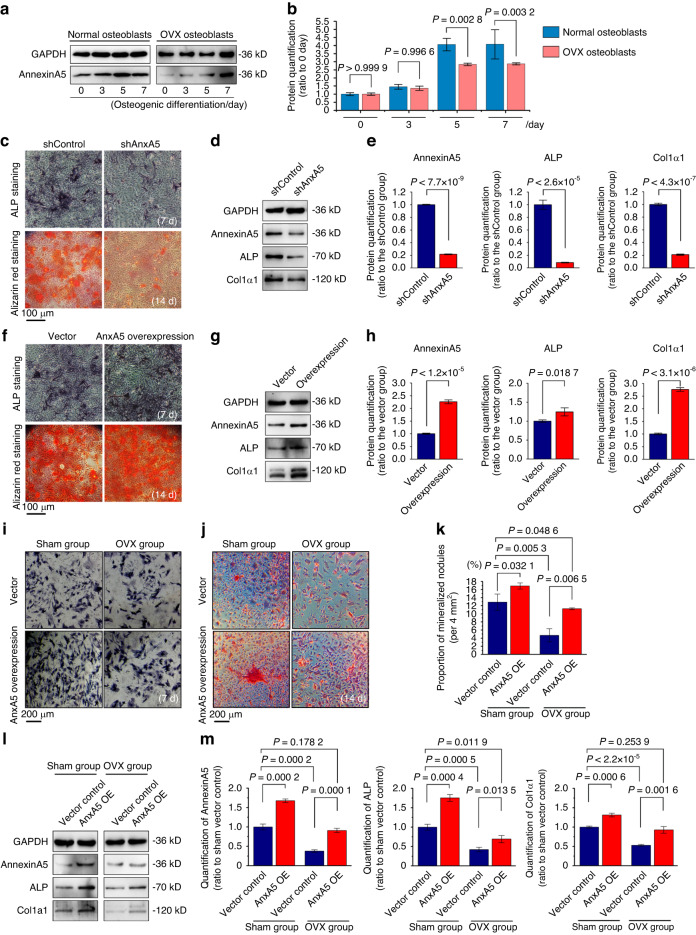
AnxA5 regulates the mineralization of osteoblasts. **a** Western blot showing the expressions of AnxA5 in normal and osteoporotic osteoblasts cultured with osteogenic media (*n* = 3). **b** Quantification of AnxA5 protein in each group (*n* = 3). **c** ALP and Alizarin red staining of MC3T3-E1 cells with or without AnxA5 knockdown after 7 or 14 days osteogenic induction. **d** Western blot showing the expressions of AnxA5, ALP and Col1α1 in MC3T3-E1 cells with or without AnxA5 knockdown. **e** Quantification of AnxA5, ALP and Col1α1 in each group (*n* = 3). **f** ALP and Alizarin red staining of MC3T3-E1 cells with or without AnxA5 overexpression after 7 or 14 days osteogenic induction. **g** Western blot showing the expressions of AnxA5, ALP and Col1α1 in MC3T3-E1 cells with or without AnxA5 overexpression. **h** Quantification of AnxA5, ALP and Col1α1 in each group (*n* = 3). **i** ALP staining showing the changes of osteogenic capacities in normal and osteoporotic osteoblasts with or without AnxA5 overexpression after 7 days osteogenic induction. **j** Representative Alizarin red staining images showing the formation of mineralized nodules in normal and osteoporotic osteoblasts with or without AnxA5 overexpression after 14 days osteogenic induction. **k** Quantitative analysis of changed mineralized nodules (*n* = 3). **l** Western blot showing the expressions of AnxA5, ALP and Col1α1 in normal or osteoporotic osteoblasts with or without AnxA5 overexpression. **m** Quantification of AnxA5, ALP and Col1α1 in each group (*n* = 3). Data in (**b**, **e**, **h**, **k** and **m**) are presented as the mean ± SD. The significant data in (**b**, **e** and **h**) were based on two-tailed Student’s *t* tests, while (**k**, **m**) were based on one-way analysis of variance (ANOVA) with Tukey’s post hoc tests

To confirm the role of AnxA5 in the mineralization of precursor osteoblasts, we first detected endogenous expression changes in AnxA5 in normal and osteoporotic osteoblasts during the osteogenic differentiation process (Fig. 3a, b). The results showed that osteogenic induction increased AnxA5 expression in normal and osteoporotic osteoblasts. Moreover, its increase in OVX osteoblasts was lower than that in normal osteoblasts after induction for 5 and 7 days. Subsequently, we transfected shAnxA5 plasmids into MC3T3-E1 cells, a preosteoblast cell line, to further identify the influence of AnxA5 on the mineralization of precursor osteoblasts (Fig. 3c). The results from ALP staining and alizarin red staining showed that AnxA5 knockdown decreased the number of ALP-positive cells and inhibited the formation of mineralized nodules. We then performed Western blotting to detect the expression changes in osteogenic differentiation-related markers after AnxA5 knockdown (Fig. 3d, e). The results indicated that the expression of ALP and Col1α1 was significantly decreased in the shAnxA5 MC3T3-E1 cells in comparison with the shControl cells. We next overexpressed AnxA5 in MC3T3-E1 cells and found that elevated AnxA5 increased ALP-positive cells and promoted the formation of mineralized nodules (Fig. 3f). Western blotting results showed that AnxA5 overexpression increased the protein expression of ALP and Col1α1 relative to that in the vector control group (Fig. 3g, h). To further confirm whether AnxA5 contributes to recovering the mineralization ability of osteoporotic osteoblasts, we used AnxA5 overexpression plasmids to enhance AnxA5 expression in normal and osteoporotic osteoblasts. ALP staining results showed that osteoblasts overexpressing AnxA5 exhibited increased ALP activity relative to those in the corresponding vector group in both normal and osteoporotic osteoblasts (Fig. 3i). Alizarin red staining was then performed, and the results indicated that overexpression of AnxA5 significantly promoted the formation of mineralized nodules in both normal and osteoporotic osteoblasts (Fig. 3j, k). Moreover, the protein expression of osteogenic differentiation markers in osteoblasts was obviously upregulated in the AnxA5-overexpressing osteoporotic osteoblasts compared with the vector group cells (Fig. 3l, m). Taken together, these data revealed that AnxA5 was essential for the mineralization of osteoblasts and could partially rescue the impaired mineralization of osteoporotic osteoblasts.

### AnxA5 promotes the adhesion of MVs to the extracellular matrix by interacting with collagen type I

**Fig. 4:**
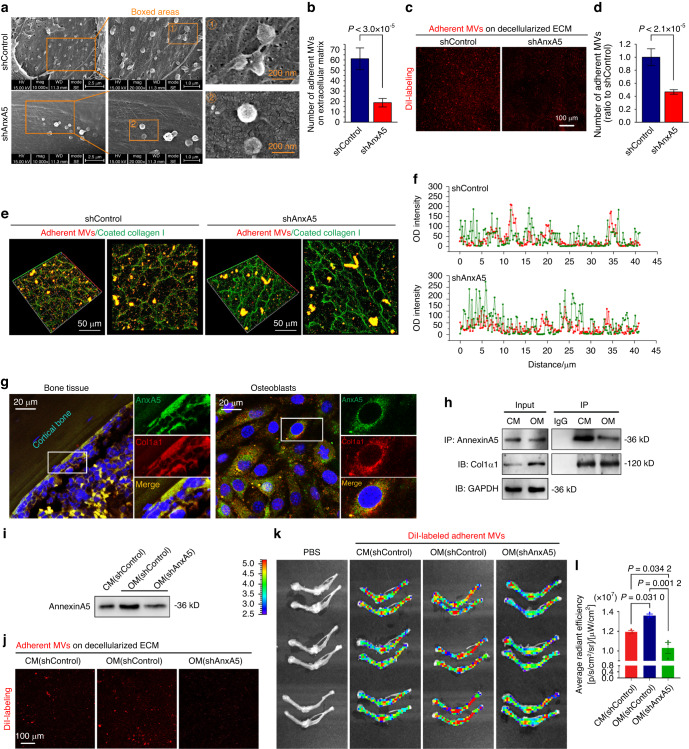
AnxA5 promotes the adhesion of MVs to ECM by interacting with collagen I. **a** SEM images showing the MVs adherent to ECM in MC3T3-E1 cells transfected with AnxA5 shRNA after osteogenic induction for 7 days. **b** Quantitative analysis showing the number changes of adherent MVs on ECM in each group (*n* = 3). **c** The adhesive capacity of MVs secreted by shAnxA5 MC3T3-E1 cells was detected after 72 h co-incubation with decellularized ECM. **d** Quantitative analysis of adherent MVs on decellularized ECM in each group (*n* = 5). **e** Immunofluorescence showing the adherent MVs of shAnxA5 MC3T3-E1 cells on the three-dimensional collagen I after incubation for 72 h (red: MVs, green: collagen I). **f** Line charts showing the colocalization of MVs and collagen I (red: MVs, green: collagen I). **g** Immunofluorescence showing the coexpression of AnxA5 and Col1α1 in normal bone tissues and primary osteoblasts (green: AnxA5, red: Col1α1, blue: Nucleus). **h** Co-IP showing the interaction between AnxA5 and Col1α1 in MC3T3-E1 cells cultured with conventional media (CM) or osteogenic induction media (OM) for 7 days. **i** Western blot showing the expressions of AnxA5 in MVs from shControl or shAnxA5 MC3T3-E1 cells cultured with OM for 7 days. **j** DiI-labeling test showing the adhesive capacities of MVs from shAnxA5 MC3T3-E1 cells after osteogenic induction for 7 days. **k** Biophotonic imaging showing the enrichment of MVs in bone tissues after equal amounts (100 mg) of DiI-labeled MVs secreted by shAnxA5 MC3T3-E1 cells were intravenously injected into mice through the tail for 12 h. **l** Quantitative analysis of the average radiant efficiency in each group (*n* = 3). Data in (**b**, **d**, **l**) are presented as the mean ± SD. The significant data in (**b**, **d**) were based on two-tailed Student’s *t* tests, while those in (**l**) were based on one-way analysis of variance (ANOVA) with Tukey’s post hoc tests

Given the close relationship between annexins and mineralization, we further aimed to investigate whether AnxA5 participated in MV-ECM adhesion, the first key step for mineralization.^35,36^ MC3T3-E1 preosteoblasts with or without AnxA5 knockdown were seeded onto the collagen type I-coated substrate, and the secreted MVs adherent to the ECM substrate were detected by SEM after 7 days of osteogenic induction (Fig. 4a, b). We observed that AnxA5 knockdown decreased the number of adherent MVs on the ECM substrate compared with that of the shControl group, indicating that AnxA5 may regulate the adhesion of MVs to the extracellular matrix. Next, DiI-labeled MVs from osteoblasts with or without AnxA5 knockdown were incubated with decellularized ECM to identify the effect of AnxA5 on MV-ECM adhesion (Fig. 4c, d). The results showed that AnxA5 knockdown reduced the number of MVs attached to the ECM, suggesting that AnxA5 is involved in the interaction between MVs and the ECM.

Collagen I, a primary component of the ECM, was shown to be involved in MV-mediated mineralization.^37,38^ We observed that the MVs were attached to the collagen type I-coated substrates (Fig. 4a); herein, we attempted to explore whether there exists an interaction between AnxA5 and collagen I. To prove this, we performed an MV-collagen I adhesion assay to determine the involvement of AnxA5 in the adherence of MVs to collagen I through CLSM (Fig. 4e), and we observed colocalization between DiI-labeled MVs (red) and collagen type I (green). Colocalization analysis with the linear immunofluorescent OD further confirmed this result (Fig. 4f). In the AnxA5 knockdown group, we found that AnxA5 ablation reduced the adhesion of MVs to collagen type I. We next used immunofluorescence double staining in bone tissues in vivo and osteoblasts in vitro to confirm the potential interaction between AnxA5 and collagen I (Fig. 4g). The results showed a colocalization between AnxA5 and collagen type I. Finally, we performed coimmunoprecipitation and found direct binding between MVs and collagen type I (Fig. 4h).

To further investigate the effect of AnxA5 on the adhesion of MVs to the ECM in vivo and in vitro, we purified MVs secreted by MC3T3-E1 cells with or without AnxA5 knockdown (Fig. 4i). The results of an in vitro MV-ECM adhesion assay demonstrated that AnxA5 knockdown resulted in a significant reduction in the number of adherent MVs attached to the ECM (Fig. 4j). Moreover, an in vivo study was performed to examine the deposition of MVs in the bone tissue of mice after intravenous tail vein injection of DiI-labeled MVs (Fig. 4k, l). In comparison to the MVs from conventional media-cultured MC3T3-E1 cells, osteogenic media induction promoted the deposition of MVs in the bone, accompanied by a higher radiant efficiency. However, MVs from MC3T3-E1 cells with knockdown of AnxA5 showed markedly lower deposition than those from normal cells, even under osteogenic induction conditions. Collectively, these results demonstrated that AnxA5 was a key membrane protein target in the binding of MVs to the ECM by interacting with collagen type I.

### AnxA5 regulates MV-mediated osteogenic mineralization via autophagy

**Fig. 5:**
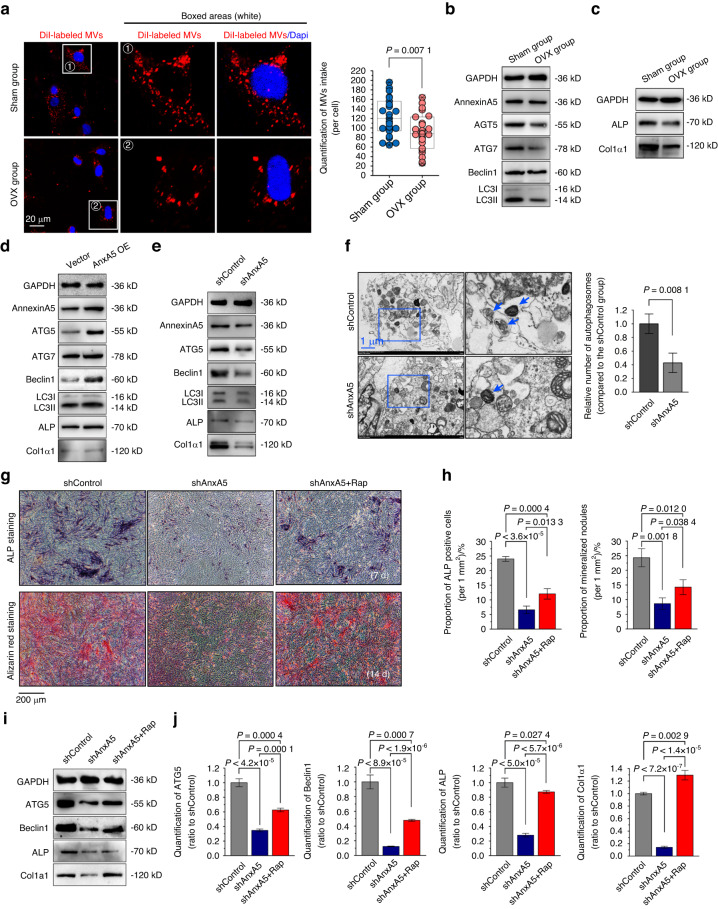
MVs mediate osteoblastic mineralization via the AnxA5-autophagy axis. **a** The uptake of MVs from normal or osteoporotic osteoblasts by BMSCs for 24 h (red: MVs, blue: nucleus). **b** Western blot showing the expressions of AnxA5, ATG5, ATG7, Beclin1 and LC3 in BMSCs treated with normal or osteoporotic MVs in osteogenic media for 5 days. **c** Western blot showing the expressions of ALP and Col1α1 in BMSCs treated with normal or osteoporotic MVs in osteogenic media for 5 days. **d** Western blot showing the expressions of AnxA5, ATG5, ATG7, Beclin1, LC3, ALP and Col1α1 in BMSCs with or without AnxA5 overexpression after 7 days osteogenic induction. **e** Western blot showing the expression of AnxA5, ATG5, Beclin1, LC3, ALP and Col1α1 in BMSCs with or without AnxA5 knockdown after 7 days osteogenic induction. **f** TEM images showing the changes of autophagy in BMSCs with or without AnxA5 knockdown. The blue arrows indicate the autophagosomes (*n* = 3). **g** BMSCs with AnxA5 knockdown were treated with autophagic activator rapamycin and cultured with osteogenic media for either 7 or 14 days. ALP and Alizarin red staining were performed to examine the expressions of osteogenesis and the formation of mineralized nodules in BMSCs. **h** Quantitative analysis of ALP positive cells (left) and mineralized nodules (right) in each group (*n* = 3). **i** Western blot showing the expressions of ATG5, Beclin1, ALP and Col1α1 in shAnxA5 BMSCs with or without rapamycin treatment after 7 days osteogenic induction. **j** Quantification of ATG5, Beclin1, ALP and Col1α1 in each group (*n* = 3). Data in (**a**, **f**, **h**, **j**) are presented as the mean ± SD. The significant data in (**a**, **f**) were based on two-tailed Student’s *t* tests, while (**h**, **j**) were based on one-way analysis of variance (ANOVA) with Tukey’s post hoc tests

To verify how AnxA5 works within precursor cells, we first performed an MV uptake assay and discovered that MVs from either normal or osteoporotic osteoblasts could be taken up by BMSCs, with far less absorption by the osteoporotic MVs than by the sham MVs (Fig. 5a).

Previous studies revealed that extracellular vesicles internalized by targeted cells could be delivered into the lysosome for degradation through the autophagy lysosome pathway and thus initialize their biological functions.^39,40^ Accordingly, we then detected changes in autophagy-related markers, including ATG5, ATG7, Beclin 1 and LC3B, in BMSCs (Figs. 5b and S3a) and osteoblasts (Fig. S4a, b). We found that these autophagy-related markers in both BMSCs and osteoblasts incubated with osteoporotic MVs showed a substantial decrease relative to those incubated with sham MVs. By using a mCherry-GFP-LC3 adenovirus double-label assay, we found changes in the occurrence of autophagy in osteoblasts (Fig. S4c–e). In normal and osteoporotic tissues, we also detected protein changes in ATG5, ATG7, Beclin 1 and LC3B (Fig. S4f–i). Moreover, we detected changes in mineralization markers, including ALP and Col1α1, in the early osteogenic differentiation of BMSCs induced by normal and osteoporotic MVs (Figs. 5c and S3b). The results indicated that the changes in mineralization capacity induced by MVs were greatly influenced by the activation of autophagy.

To further identify the effect of AnxA5 on autophagy, we transfected AnxA5 overexpression or knockdown plasmids into BMSCs, and the changes in autophagy and osteogenic mineralization in the early osteogenic differentiation of BMSCs (7 days of osteogenic induction) were examined by Western blotting (Fig. 5d, e). The results showed that compared with the vector control, AnxA5 overexpression upregulated the expression of the autophagy-related markers ATG5, Beclin1 and LC3, as well as the osteogenic differentiation markers ALP and Col1α1 (Figs. 5d and S3c). Conversely, AnxA5 knockdown attenuated autophagy levels, with reduced expression of ATG5, Beclin1 and LC3, and impaired osteogenic differentiation, with reduced expression of ALP and Col1α1 (Figs. 5e and S3d). We then further explored the occurrence of autophagy by TEM, and the results showed that AnxA5 knockdown decreased the formation of autophagosomes (Fig. 5f). In addition, rapamycin (Rap), an autophagy activator, was applied to BMSCs with AnxA5 knockdown to identify whether AnxA5 mediated mineralization via autophagic flux. The results based on ALP and alizarin red staining indicated that AnxA5 knockdown inhibited the formation of mineralized nodules, while Rap treatment rescued the loss of mineralized nodules (Fig. 5g, h). From the Western blotting results, Rap treatment enhanced the AnxA5 knockdown-impaired autophagy markers ATG5 and Beclin1 and the osteogenic differentiation markers ALP and col1α1 (Fig. 5i, j). Taken together, these data indicated that AnxA5 regulates MV-mediated osteogenic mineralization via autophagy.

### AnxA5 plays a protective role in resisting bone loss in osteoporotic mice

**Fig. 6:**
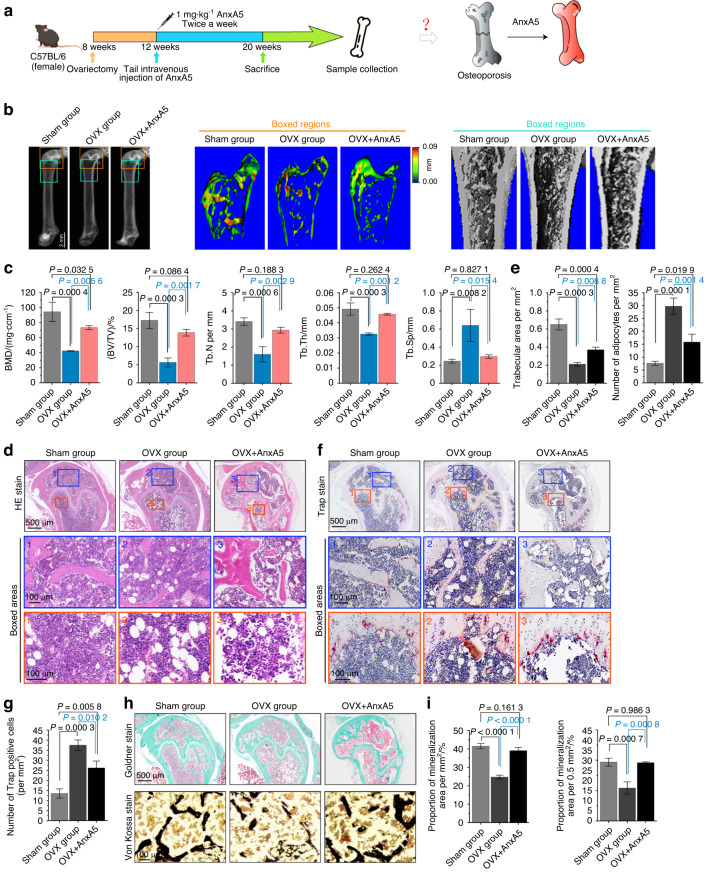
AnxA5 contributes to recovering bone loss in osteoporosis in vivo. **a** Schematic illustration showing the experimental design. **b** Micro-CT images of the distal femurs from normal mice, OVX mice, and OVX mice treated with AnxA5 recombinant protein. **c** Quantitative micro-CT parameters of bone mineral density (BMD), trabecular bone volume per tissue volume (BV/TV), trabecular number (Tb.N), trabecular thickness (Tb.Th) and trabecular separation (Tb.Sp) (*n* = 3). **d** H&E staining showing the morphological changes of distal femurs in each group. **e** Quantitative analysis confirming the effect of AnxA5 on the formation of trabeculae and the production of adipocytes (*n* = 3). **f** Trap staining showing the formation of osteoclasts at distal femurs in each group. Osteoclasts are stained red. **g** Quantitative analysis of Trap-positive osteoclasts (*n* = 3). **h** Goldner and Von Kossa staining showing the mineralized bones at distal femurs in each group. Mineralized bones are stained green in Goldner staining and stained black in Von Kossa staining. **i** Quantitative analysis of the mineralized bones confirming the effect of AnxA5 on bone mineralization (*n* = 3). Data in (**c**, **e**, **g** and **i**) are presented as the mean ± SD, and the significant data were based on one-way analysis of variance (ANOVA) with Tukey’s post hoc tests

To determine whether AnxA5 has therapeutic effects on osteoporosis, we intravenously injected AnxA5 recombinant protein into the tails of normal and osteoporotic mice (Fig. 6a). Twelve hours after injection, we detected the potential toxicity of AnxA5 to visceral organs and found that no toxicity appeared in visceral organs in the early stages after injection (Fig. S5a); moreover, we tested the indicators in the serum of mice after injection of AnxA5 for two months and found that the indicators in the serum were normal (Fig. S5b). These results indicated that the injection of AnxA5 did not cause significant toxicological changes in mice. Three-dimensional (3D) reconstructions of microcomputed tomography (micro-CT) scans and quantitative morphometric analyses of the femur were then performed to detect the changes in osteoporotic bone after injection of AnxA5 for two months (Fig. 6b, c). We found that compared with the sham mice, osteoporotic mice showed a lower bone mass (BMD and BV/TV) and poorly organized trabecular architecture (Tb.N, Tb.Th, and Tb.Sp), which is consistent with the known pathological characteristics of osteoporosis.^1,41^ After injection with AnxA5, the osteoporotic bone loss in ovariectomized mice was strikingly relieved. From histology, we found that AnxA5 injection retained the number of cancellous bone in osteoporotic mice and alleviated the degree of adiposity in the bone marrow of osteoporosis (Fig. 6d, e). We then performed TRAP staining to observe the changes in bone resorption by indicating the distribution of osteoclast activities (Fig. 6f, g). The results revealed that AnxA5 injection impaired the enhancement of osteoclast activities in osteoporotic mice. Finally, by using Goldner and von Kossa staining, we detected mineralization changes in bone (Fig. 6h, i). The results indicated that the reduced proportion of mineralization areas in the OVX group exhibited a remarkable increase after AnxA5 injection. Taken together, these findings demonstrated that AnxA5 contributes to attenuating bone loss and slowing the progression of osteoporosis.

## Discussion

The skeleton undergoes remodeling throughout life, where coordinated bone formation and bone resorption contribute to maintaining bone homeostasis.^42–44^ Serving as the primary cell type responsible for bone formation, osteoblasts of mesenchymal origin are involved in the process of osteogenesis.^45,46^ Osteoblasts generally undergo a series of processes to build the bone matrix as follows: cell proliferation and differentiation with elevated expression of osteogenic markers, including Runx2, ALP and collagen I, as well as extracellular matrix synthesis and MV-mediated extracellular matrix mineralization, thus leading to bone formation.^47,48^ Reduced osteoblast activity and excessive impairment of bone matrix accumulation result in the loss of bone mass, destruction of bone microarchitecture, and the final occurrence of osteoporosis.^49,50^ To date, as a vital subtype of extracellular vesicles that serve as intercellular communicators, MVs have been identified to play an essential role in physiological mineralization.^13–19^ Moreover, the importance of MVs in the process of osteoporosis has been recently reported,^51,52^ but their deposition characteristics have not yet been investigated. MVs were initially discovered in calcified cartilage by using an electron microscope^11^ and were then detected in other calcifying tissues, including dentin^14^ and blood vessels.^28^ In this study, MV budding from the membrane of osteoblasts was observed in the osteoid region of the bone matrix in vivo (Fig. 1a). Notably, in contrast to that from the normal bone tissue, adherent MVs secreted by osteoblasts from OVX bone showed a major decrease both in vivo and in vitro (Fig. 1b–f). Moreover, the pathological process of osteoporosis changes the intrinsic characteristics of MVs, including components (Fig. 2c), mineralization capacity (Figs. 3c–m), adhesion ability (Fig. 4) and deposition of bone matrix (Fig. 6b–i). These results not only indicated the potent role of MVs in the mineralization of osteogenic progenitor cells/mesenchymal stem cells but also revealed the intrinsic differences in bone masses in both normal and osteoporotic tissues from the perspective of secreted MVs. Candidates such as AnxA5 extracted from MV components provide potential targets to prevent bone loss in osteoporosis.

During mineralization, MVs secreted by osteoblasts can either adhere to the ECM (adherent MVs) or be released into the blood (released MVs). Increasing evidence has shown that osteoblast-derived MVs under different physiological and pathological conditions show different distributions, compositions and activities.^53–55^ A previous in vitro mineralization model identified that during the early stage of osteogenic differentiation, adherent MVs accounted for the vast majority of all MVs.^54^ However, the proportion of released MVs exhibited a slight increase with the extension of osteogenic induction time. Moreover, these two kinds of MVs exhibited distinguishable compositions, which determined their different biological functions. In this study, our results showed that adherent MVs exhibited strong adhesion and mineralization relative to released MVs (Fig. 1g–m). This finding might be due to the specific abundant mineralized components in adherent MVs,^56^ which was confirmed by Chaudhary et al.^54^ and Kapustin et al.^55^ In addition, we found that adherent MVs secreted by osteoporotic osteoblasts were significantly decreased while released MVs into serum were increased compared with those of normal osteoblasts (Fig. S1). Together, these results not only indicated the differences in the number of MVs from healthy subjects and subjects with osteoporosis but also demonstrated their differences in biological roles, thus providing new evidence for understanding the versatility of MVs and providing a new perspective on the etiology of osteoporosis from the perspective of MVs.

MVs can be secreted either under normal physiological conditions or pathological processes.^57^ Proteomic research showed that the composition of matrix vesicles was varied when osteoblasts were exposed to different external stimuli or under different pathological conditions.^58,59^ The fate and biological function of MVs are generally determined by their specific composition.^60^ Annexin A is a calcium-dependent phospholipid binding protein and is widely distributed in a variety of tissues and cells.^61,62^ To date, twelve members of the annexin A family (AnxA1-AnxA11, AnxA13) are known to exist in vertebrates, and the majority of them can be found in MVs with high expression levels.^53,58^ Balcerzak et al. characterized the proteome of MVs isolated from femurs of chicken embryos and found an abundant enrichment of annexins in MVs, including AnxA1, AnxA2, AnxA4, AnxA5, AnxA6, and AnxA11, among which AnxA5 had the highest expression level.^63^ Xiao et al. also discovered high expression of AnxA2, AnxA5 and AnxA6 in MVs released by mineralized MC3T3-E1 cells.^20^ Here, we further explored the changes in the annexin A family from normal and osteoporotic MVs. As shown in Fig. 2a–d, the expression levels of AnxA1, AnxA2, AnxA4, AnxA5, AnxA6, AnxA7 and AnxA11 were all decreased in osteoporotic MVs relative to normal MVs. Moreover, AnxA5, AnxA2, and AnxA6 showed significantly differential expression among the top three genes (Fig. 2e–i). This result prompted us to focus on the role of AnxA5 in the mineralization of bone tissues.

As one of the most highly enriched annexin members on the surface of MVs, AnxA5 has been reported to regulate the influx of calcium,^21^ trigger osteogenic-related signal transduction,^58^ and even potentially impact biomineralization.^64^ In skeletal development, a report revealed that AnxA5 could regulate the cell proliferation-apoptosis axis by binding to phospholipids, thereby affecting the growth and development of cartilage and bone.^65^ In bone modeling, evidence has been accumulated to indicate its importance. Haut Donahue et al. demonstrated that AnxA5 can sensitively respond to oscillating fluid flow and mediate flow-induced calcium signaling in osteoblasts, which would be inhibited by treatment with blocking antibodies or a pharmacological inhibitor, K201 (JTV-519).^66^ Genetos DC et al. found that knockdown of AnxA2 and AnxA5 in preosteoblasts could significantly reduce the expression of Col1α1, Runx2, BGLAP and other osteogenic protein markers in osteoblasts, thus inhibiting cell proliferation, differentiation and bone formation.^64^ Given the enrichment and osteogenic capacity of the annexin A family in MVs,^20^ AnxA5, with its relatively high expression, showed a major impact on bone mineralization of osteogenic progenitor cells (Fig. 3a–h), and its overexpression further enhanced the mineralization of osteoporotic osteoblasts both in vitro (Fig. 3i–m) and in vivo (Fig. 6b–i). This evidence identified AnxA5 as a core target in the role of MVs in mediating bone formation and mineralization and provided a potential applicable option in bone mass maintenance in the pathological progression of osteoporosis.

An appropriate ‘calcifiable’ matrix is necessary for hydroxyapatite crystal growth and deposition. When preosteoblasts experience osteoblastic differentiation, dissociative MVs with mineralization-specific components, including calcium and phosphorus ions, are recruited to the peripheral extracellular matrix space and subsequently attach to the extracellular matrix, releasing calcium/phosphate components to facilitate mineralization.^17,67^ The components and mineralization properties of MVs are largely dependent on the intracellular composition of the parent cells without denying that the surrounding microenvironment may also make a difference.^68^ MVs for matrix mineralization have been widely recognized to first attach to the extracellular matrix, which is directly influenced by the proteins constituting the outer membrane of MVs. Kim et al. reported that there are several surface receptors that mediate cell and MV interactions in growth plate chondrocytes, including integrin receptors (α1, α2, α5, α6, αV, β1 and β5) and nonintegrin receptors, such as annexin V and CD44.^29^ Herein, we found a role for MV-derived AnxA5 in extracellular matrix adhesion (Fig. 4a–d). Previous studies have confirmed that MVs and calcium and phosphorus crystals were present within and between type I collagen fibers, the main organic component of the extracellular matrix of growth plate cartilage and bone mineralizing areas.^69,70^ We confirmed that MVs could attach to collagen type I by direct binding through AnxA5 (Fig. 4e–h), which was the most critical step for MVs to act on osteogenic progenitor cells and play a role in mineralization. In addition to the binding of collagen type I, AnxA5 could also interact with other molecules, such as collagen types II and X.^37,71^ These bindings together determined the adhesive capacity of MVs and their influence on the mineralization of bone tissues.

MVs enhanced the osteogenic differentiation of BMSCs and promoted the mineralization capacity in this study. Notably, once AnxA5 was knocked down, the mineralization ability of BMSCs exhibited a decrease compared with that of the sham group (Fig. 5). The reasons may be due to the interaction of annexins with proteins present on the surface of cells, such as phosphatidylserine (PS) and integrins.^72^ Stimulation of calcium and phosphate promotes the exposure of PS on the cell membrane, followed by interactions with calcium-binding protein S100A9 and AnxA5 and induces the uptake of MVs into BMSCs, thus driving mineralization.^73^ AnxA2 has been previously reported to bind with integrin α5 to promote flow-induced endothelial activation.^72^ To further examine the mechanisms of MV-mediated mineralization, changes in autophagy, we identified a mechanism involved in osteogenic differentiation, in this study. Previous reports have demonstrated that autophagy plays a crucial role in promoting osteoblastic mineralization and regulating bone homeostasis.^74,75^ Here, we found that attenuated autophagy is closely related to the occurrence of osteoporosis, accompanied by reduced expression of AnxA5 (Fig. 5). Zhang et al. found that TRIM21 facilitated the translocation of ANXA2 toward the plasma membrane and subsequently led to cell differentiation by inducing autophagy in osteosarcoma cells.^76^ AnxA5 was also found to participate in autophagic regulation.^24^ We showed that MVs induced autophagy via AnxA5 and thus promoted osteogenic differentiation in BMSCs (Fig. 5). Collectively, we showed that MVs could regulate the osteogenic differentiation of BMSCs through AnxA5-mediated autophagy.

There are some limitations in the current study. First, regarding the contribution of AnxA5 in MVs to mineralization, we revealed the effect of MV-derived AnxA5 on the mineralization of osteogenic progenitor cells/mesenchymal stem cells in vitro and indicated its potential role in the positive deposition of bone matrix in osteoporotic mice. However, there are many other proteins in MVs that are likely to work directly or synergistically with AnxA5 to achieve the mineralization of bone tissues. Therefore, the contribution of other participants of MVs in mineralization must be considered when applying MVs for bone protection in practice. Second, we used recombinant AnxA5 protein instead of AnxA5-expressing MVs to treat osteoporosis due to the difficulty of AnxA5-MV collection. Thus, we did not directly demonstrate the role of MV-derived AnxA5 in protecting against bone loss in osteoporosis. It may be necessary to design new methods that could directly obtain a sufficient amount of AnxA5-expressing MVs to demonstrate their effectiveness in future work, although we have already observed the effect of AnxA5 on promoting mineralization. We could infer that if AnxA5-expressing MVs are used, the mineralization effect should be better because AnxA5-expressing MVs not only carry the annexin family, including AnxA5 but also provide other favorable proteins for mineralization and mineralized components, including calcium and phosphorus. Third, regarding the drug delivery system, we used traditional tail intravenous injection but not local injection or a bone-targeted drug delivery system. Local injection seems to be an optimized alternative option for AnxA5 administration due to its high utilization efficiency, but the proper dosages that do not lead to excessive deposition are generally hard to determine.^77^ A bone-targeted drug delivery system is an advanced optimized method that enables drugs to accumulate specifically in the target skeleton tissues, enabling the improvement of their therapeutic efficacy.^78,79^ It is expected to achieve the desired effect with a smaller dose of the drug and relatively minor side effects. However, this system also has some disadvantages, such as a complicated preparation process and uncertain biocompatibility. In the current study, we chose traditional tail intravenous injection, a commonly used drug delivery system due to its high operability and precise control over drug doses.^80–82^ Even in the absence of local or targeted delivery, we also observed the effect of AnxA5 on the mineralization of bone tissues. Thus, this result indicated the effectiveness of MV-derived AnxA5 in mineralization and inferred that its effectiveness would be more significant when based on a more effective drug delivery system.

**Fig. 7:**
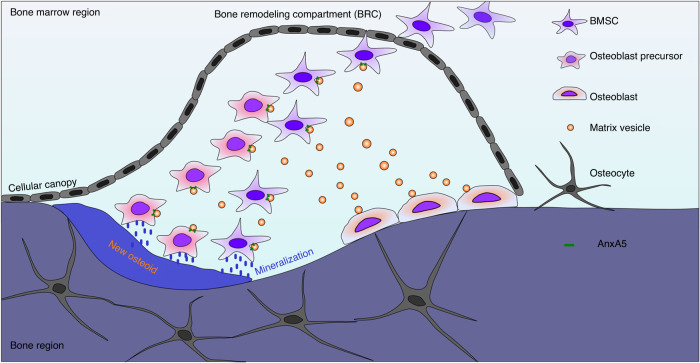
Schematic illustration demonstrating the role of AnxA5 in matrix vesicle-mediated mineralization. During the bone remodeling process, osteoblasts secrete numerous matrix vesicles. These vesicles loaded with AnxA5 could be prominently taken up by BMSCs or osteoblast precursor cells, leading to osteogenic differentiation and mineralization

In summary, we found decreased MVs in osteoporotic mice, accompanied by attenuated mineralization characteristics. The impaired mineralization capacity of MVs was due to the decrease in AnxA5. MV-derived AnxA5 participates in bone mineralization by regulating MV attachment to the extracellular matrix and stimulating osteogenic differentiation of peripheral osteogenic progenitor cells/mesenchymal stem cells via autophagic induction (Fig. 7). Collectively, these findings may provide new insights into understanding matrix vesicles. The role of MV-derived AnxA5 in mineralization regulation provides new clues for the prevention of bone loss in osteoporosis.

## Materials and methods

### Animals

Before animal usage, the protocol was first reviewed and approved by the IRB at the Institute of Biomedical Engineering, West China School of Basic Medical Sciences & Forensic Medicine (K2021015). Female C57BL/6 mice (6 weeks, 18 ~ 22 g) were purchased from Chengdu Dossy Biological Technology Co., Ltd. (Chengdu, China). The mice were housed under specific pathogen-free conditions with a constant temperature of 25 °C and a 12 h light/dark cycle. The cages were regularly cleaned and supplied with plenty of food and water. The mice were fed an alfalfa-free diet for at least 1 week before the experiment.

### OVX mouse model

The female C57BL/6 mice were randomly divided into three groups: sham group (sham), OVX group (OVX), and AnxA5-treated OVX group (OVX + AnxA5). For the OVX group, ovariectomy was performed as previously described.^41^ Briefly, the mouse was anesthetized with 1.5% isoflurane (R510-22, RWD Life Science Co., Ltd., Shenzhen, China) at a rate of 0.5–1.0 L·min^−1^ and then placed on a 37 °C thermostatic heating plate in a prone position. After the hair was shaved and the dorsal area was sterilized with 75% alcohol, an approximately 1 cm cut was made in the middle dorsal skin, and the ovary was thoroughly excised with scissors. The wound was sutured and wiped with 2% iodophor disinfectant. The bilateral ovaries were both removed as described above. For the sham group, the same procedure was conducted without removing the ovaries. For the OVX + AnxA5 group, one month after the ovariectomy surgery, the mice were intravenously injected with 1 mg·kg^−1^ AnxA5 twice a week for 8 weeks. The mice in each group were sacrificed 3 months after the surgery. The serum and bilateral femurs and tibias were collected for subsequent in vivo and in vitro analyses.

### Cell isolation and culture

The preosteoblast cell line MC3T3-E1 (Jennio Biotechnology Co., Ltd., Guangzhou, China) was cultured with alpha minimal essential media (α-MEM, Gibco, California, USA) with 10% fetal bovine serum (FBS, Aoke, Chengdu, China) and 1% penicillin‒streptomycin solution in a humidified atmosphere of 5% CO_2_ at 37 °C.

C57BL/6 mice were used in this study to isolate primary osteoblasts and BMSCs. BMSCs were isolated from the bone marrow of the femurs and tibias according to the protocol provided.^83^ Briefly, mice were anesthetized and killed, and the long bones were immediately dissected under sterile conditions and placed in α-MEM with 1% penicillin‒streptomycin (PS, SV30010, HyClone, Utah, USA). The soft tissue and periosteum attached to the bone were thoroughly removed, and the epiphyses were cut. The marrow in the bone cavities was then gently flushed out with α-MEM with 1% PS. After centrifugation at 1 500 × *g* for 5 min, the marrow pellets were resuspended in fresh complete α-MEM and transferred to cell culture flasks. The media were replaced to remove nonadherent cells after 3 h, 24 h and 48 h of culture. The remaining adherent cells, BMSCs, were cultured until reaching 90% confluence. Cells at passages 2–4 were used in the experiment. The above marrow-depleted bones were then used for osteoblast isolation as described previously.^84^ Briefly, the bones were excised into approximately 1–2 mm^3^ small pieces and digested with 0.05% trypsin-EDTA (25200056, HyClone, Utah, USA) for 20 min, followed by 3 washes with phosphate-buffered saline (PBS, SH30256, HyClone, Utah, USA). Subsequently, collagenase IA (1 mg·mL^−1^; C8140, Solarbio, Beijing, China) digestion was performed for 60 min and repeated twice. The collagenase solution was collected each time to obtain osteoblasts by centrifuging at 1 000 r·min^−1^ for 5 min. The isolated osteoblasts were then cultured with fresh complete α-MEM media at 37 °C in a 5% CO_2_ incubator. For purification, the digested cells were cultured for 20 min before transferring the nonadherent cells to a new culture flask. Passages 3–5 were used for the follow-up experiment.

### Antibodies and reagents

Detailed information about the antibodies used for Western blotting analysis and immunofluorescence staining is shown in Table S1. Rapamycin (Rap, 100 nmol·L^−1^; V900930, Sigma-Aldrich, Missouri, USA) was used to induce autophagy by inhibiting the activity of mTOR in this study. Osteogenic induction media (OM) was prepared by adding additional ascorbic acid (100 μmol·L^−1^; A7506, Sigma-Aldrich, Missouri, USA), dexamethasone (10 nmol·L^−1^; D4902, Sigma-Aldrich, Missouri, USA) and β-glycerophosphate (10 mmol·L^−1^; G9422, Sigma-Aldrich, Missouri, USA) to the complete α-MEM media.^28^

### Plasmid and transfection

For AnxA5 knockdown, MC3T3-E1 cells/primary osteoblasts were transfected with the shRNA plasmid (Tsingke Biotechnology Co., Ltd., Beijing, China) targeting AnxA5 toward the sequence of 5ʹ-CCGGCGTGAAGTCTATTCGGAGCATCTCGAGATGCTCCGAATAGACTTCACGTTTTTG-3ʹ using Lipofectamine 8000 (Beyotime, China) according to the manufacturer’s protocol (named the shAnxA5 group). The shRNA plasmid with the sequence 5ʹ-CCGGGGTTCTCCGAACGTGTCACGTCTCGAGACGTGACACGTTCGGAGAACCTTTTTGAATT-3ʹ served as the negative control (named the shControl group). After 48 h of incubation, complete medium containing 5 μg·mL^−1^ puromycin (ST551, Beyotime, Shanghai, China) was used to select shAnxA5 stably transfected cells. For AnxA5 overexpression, the plasmid was constructed by Shanghai GenePharma Co., Ltd. (named the AnxA5 overexpression group). The vector plasmid served as the negative control (named the vector group). MC3T3-E1 cells or osteoblasts were transfected with the plasmids using Lipofectamine 8000 (C0533, Beyotime, Shanghai, China) according to the manufacturer’s protocol. After 48 h of transfection, G418 (500 μg·mL^−1^; ST081, Beyotime, Shanghai, China) was used to select stably transfected cells. Once the cells reached confluency, they were collected for final transfection efficiency identification by Western blotting analysis.

### Isolation of matrix vesicles

For bone-derived MVs, femurs and tibias were dissected, and the attached muscles and periosteum were cleaned. Marrow-depleted bones were then crushed to powder in liquid nitrogen, followed by digestion with collagenase IA (100 U·mL^−1^, in Hank’s balanced salt solution) for 3 h in a shaking incubator at 37 °C with a shaking speed of 200 r·min^−1^. The solution was then collected and filtered with a 0.48 μm filter. The MVs were finally purified using an exosome isolation kit (EX010, Shanghai Gefan Biotechnology Co., Ltd., Shanghai, China) according to the manufacturer’s protocol.

For cell-derived MVs, once the cell confluency reached approximately 80%, the growth media were replaced with α-MEM with 10% MV-free FBS and 1% PS. The matrix vesicles from the ECM were isolated after 48 h of incubation according to the protocol previously described.^33^ Briefly, the growth media were removed, and the remaining cells were washed 3 times with PBS. Collagenase IA (0.8 mg·mL^−1^; C8140, Solarbio, Beijing, China) was then used to digest the ECM and release the MVs. After 4 h of incubation at 37 °C, the digestion solution was collected and centrifuged as follows: 300 × *g* for 20 min (to discard pelleted cells), 3 000 × *g* for 20 min (to discard pelleted cell debris), 10 000 × *g* for 30 min (to discard pelleted apoptotic bodies and microvesicles), and 100 000 × *g* for 120 min to obtain the MV pellet. Finally, the pellet was resuspended in 100 µL of PBS and either used immediately or stored at −80 °C.

### Protein extraction, quantification and Western blotting

Collected cells, MVs or preground bone tissues were lysed with RIPA lysis buffer with protease inhibitor, phosphatase inhibitor and phenylmethylsulfonyl fluoride for 30 min on ice. The protein was extracted by centrifugation at 14 000 × *g* for 10 min at 4 °C, followed by determination of the protein concentration using a BCA protein assay kit (P0012, Beyotime, Shanghai, China). Western blot assays were performed as previously described.^85^ Equal amounts of protein (20 µg) from each sample were subjected to electrophoresis on 8%/10%/12% sodium dodecyl sulfate‒polyacrylamide gels and then transferred to a polyvinylidene difluoride (PVDF) membrane. After the membrane was blocked with 5% skim milk at room temperature for 2 h, it was incubated overnight with specific primary antibodies (diluted in 5% skim milk) on a roller bank at 4 °C and treated with the corresponding HRP-conjugated secondary antibodies for 1–2 h at room temperature. The results were finally visualized and analyzed with enhanced chemiluminescence using a Molecular Image® Chemi-DocTM XRS+ system with Image LabTM Software. Glyceraldehyde-3-phosphate dehydrogenase (GAPDH) served as the internal control in this study.

### Immunofluorescence of cells/tissues

For the immunofluorescence of cells, 1 × 10^5^ cells of each group were seeded onto a coverslip placed in 24-well plates and cultured with osteogenic induction media for 7 days. The cells were then rinsed three times with PBS and fixed with 4% paraformaldehyde for 15 min. After the cells were blocked with 5% bovine serum albumin (BSA, BS114, Biosharp, Hefei, China) for 30 min at room temperature, they were incubated with primary antibodies (1:500, diluted in 5% BSA) overnight at 4 °C. The corresponding fluorochrome-labeled secondary antibodies (1:1 000, diluted in 1% BSA) were applied to the cells for 60 min after washing with PBS three times. The nuclei were subsequently stained with 4′, 6-diamidino-2-phenylindole (DAPI, 1:800, C1002, Beyotime, Shanghai, China) for 10 min and rinsed five times with PBS gently. The images were captured using a Zeiss (LSM710, Oberkochen, Germany) confocal microscope.

For the immunofluorescence of bone tissues, the paraffin sections were deparaffinized in xylene and hydrated in graded ethanol, followed by immersion in heated sodium citrate buffer (10 mmol·L^–1^, pH 6.0) for antigen retrieval. Subsequently, the samples were blocked, incubated with antibodies and stained for nuclei as described above.

### Coimmunoprecipitation (co-IP) of AnxA5

The coimmunoprecipitation (co-IP) assay was performed as described previously.^86^ Briefly, 1 mg of total protein from the lysates was incubated with 1 μL of primary antibodies against AnxA5 overnight at 4 °C. Then, 20 µL of A/G agarose beads (P2012, Beyotime, Shanghai, China) was added to the above solution for 2 h of incubation with gentle vibration at 4 °C. After three washes with RIPA solution, the beads were precipitated by centrifuging at 5 000 × *g* for 1 min at 4 °C. The protein was then eluted with 2× loading buffer by boiling for 8 min. The following immunoblotting assay was similar to the protocol of Western blotting described above.

### Proteomic analysis

MVs (1 mg in PBS) secreted by normal or osteoporotic individuals were purified as described above and were then used for LC–MS/MS analysis. The analysis was processed by KangChen Biotech Co., Ltd. The GO analysis was performed based on differentially expressed proteins.

### Alizarin red staining

The cells reaching confluency were cultured with osteogenic induction media for 7 days or 14 days and were then fixed with 95% alcohol for 15 min. Next, the cells were washed three times and stained with 2% Alizarin red solution (pH 4.2, C0138, Beyotime, Shanghai, China) for 30 min at room temperature. After the excess dye was removed and the cells were rinsed five times with ddH_2_O, the mineralized nodules were observed by an inverted microscope (CK2, Olympus, Tokyo, Japan).

### Alkaline phosphatase staining

The cells reaching confluency were cultured with osteogenic induction media for 7 days. ALP staining was performed using a kit (C3206, Beyotime, Shanghai, China) according to the manufacturer’s protocol. Briefly, cells were fixed with 4% paraformaldehyde for 15 min and subsequently coincubated with prepared working solution for 0.5–1 h at room temperature. The reaction was terminated by rinsing the cells 3 times with ddH_2_O.

### Alkaline phosphatase activity assay

The cells were cultured with osteogenic media for 7 days, lysed with RIPA lysis buffer without the addition of phosphatase inhibitor on ice and centrifuged at 14 000  r·min^−1^ for 10 min. ALP activity in the supernatant was measured using *p*-nitrophenol phosphate substrate supplied in an ALP assay kit (P0321S, Beyotime, Shanghai, China). The absorbance was measured at 405 nm, and the final ALP activity was normalized to the protein concentration.

### Nanoparticle tracking analysis

The MV pellet was resuspended in 100 μL of PBS, and the size and concentration were subsequently measured by nanoparticle tracking analysis (NTA) using a ZetaView instrument (PMX120, Particle Metrix, Munich, Germany). Data acquisition and analysis were performed using Zetaview Analytical Software.

### Transmission electron microscopy (TEM)

For observation of the morphology of MVs, the samples resuspended in PBS were dropped on a copper mesh for 2 min of adsorption. When the solution was removed using filter papers, 2% phosphotungstic acid solution was applied to the copper grid for 10 min at room temperature to perform negative staining. After the staining solution was removed and the sample was dried, the MVs were observed and captured using TEM (HT7800, Hitachi High-Tec Co., Ltd., Tokyo, Japan) at 80 kV.

For observation of the MVs deposited in bone, the dissected distal femurs were fixed in 2.5% glutaraldehyde for 12 h and decalcified thoroughly with EDTA solution (G1107, Servicebio, Wuhan, China). After three rinses with PBS, the tissues underwent gradient acetone dehydration, 100% acetone-Epon 812 embedding, and ultrathin sectioning in sequence. The samples were then double‐stained with uranyl acetate-lead citrate, and the MVs were detected by TEM.

For determination of the formation of autophagosomes, the confluent cells in each group were collected and centrifuged at 1 500 r·min^−1^ for 5 min. Then, the cell pellets were fixed, dehydrated, embedded and sliced as described above. The autophagosomes were observed by TEM.

### Scanning electron microscopy (SEM)

For observation of the MVs adhered to the ECM, the cells were fixed with glutaraldehyde fixative, especially for electron microscopy (2.5%), overnight at 4 °C after 7 days of osteogenic induction culture. After three rinses with ddH_2_O, the samples were dehydrated using graded ethanol (30%, 50%, 70%, 85%, 95%, 100%, 100%) for 15 min each time. The dehydrated samples were then placed in a critical point dryer for CO_2_ drying, sputtered with gold and observed with SEM (Phenom Prox, Phenom-World, Eindhoven, Netherlands).

### MV uptake and coculture assays

BMSCs were seeded on 10 mm glass coverslips placed in 24-well plates and cultured overnight. Equal amounts of purified MVs (1 × 10^10^) were labeled with DiI (C1036, Beyotime, Shanghai, China) according to the manufacturer’s instructions and added to the growth media in each well. After 24 h of coincubation, the uptake of MVs by BMSCs was analyzed by confocal microscopy. The nuclei were stained with DAPI after 15 min of fixation in 4% paraformaldehyde.

For determination of the effect of MVs on the osteogenic differentiation of BMSCs, 5 × 10^5^ cells per well BMSCs were seeded into 6-well plates and cultured overnight. The cells were then treated with fresh growth medium containing equal concentrations of MVs, which was replaced every other day. Total protein was collected for further Western blotting analysis after 5 days of coincubation.

### In vitro MV-ECM/collagen I adhesion assay

For the in vitro MV-ECM adhesion assay, ECM was prepared on coverslips placed in 24-well plates. MC3T3-E1 cells were seeded on coverslips, cultured to confluence, and then incubated with osteogenic induction media for 7 days. The cells were then rinsed three times with PBS and decellularized by treatment with 20 mmol·L^−1^ NH_4_OH/0.25% Triton X-100 at room temperature for 3 min. The cell debris was removed, and the remaining decellularized ECM was washed three times with PBS for subsequent use. Equal amounts of DiI-labeled MVs (1 × 10^10^) were diluted in complete growth media and added to the prepared decellularized ECM. After 72 h of incubation at 37 °C, the anchored MVs on the ECM were detected by fluorescence microscopy (Olympus, Tokyo, Japan).

For the MV-collagen I adhesion assay, glass coverslips in 24-well plates were coated with type I collagen at a concentration of 2 mg·mL^−1^ with 0.006 mol·L^−1^ acetic acid and 0.1 mol·L^−1^ NaOH and placed at room temperature for 4 h. Equal amounts of DiI-labeled MVs were then added to the collagen I-coated coverslips and cocultured for 72 h. Collagen I was then determined using the IF assay described above, and the MVs adherent to the ECM were also observed by confocal microscopy.

### Micro-CT analysis

The dissected tibias and femurs were fixed with 4% paraformaldehyde for 24 h at room temperature and subsequently scanned ex vivo using a micro-CT scanner (mCT50, Scanco Medical, Bassersdorf, Switzerland). Three-dimensional reconstructions of tibias and femurs were performed with a micro-CT system. The bone morphometric parameters, including bone mineral density (BMD), ratio of bone volume to tissue volume (BV/TV), trabecular number (Tb.N), trabecular thickness (Tb.Th) and trabecular separation (Tb.Sp), were analyzed.

### Histological staining and analysis

The isolated tibias and femurs were fixed in 4% paraformaldehyde for 24 h and then decalcified with EDTA solution in a constant rotating shaker at room temperature. After dehydration with gradient ethanol, the bone tissues were embedded in paraffin and cut into 5–7 μm sections. The sectioned tissues were placed on slides and dried overnight for subsequent histological analysis, including hematoxylin and eosin staining (H&E), Goldner staining and TRAP staining. The stained tissues were scanned using a digital slice scanner (VS200, Olympus, Tokyo, Japan), and the images were captured using CaseViewer Software.

### Biophotonic imaging analysis

DiI-labeled MVs (100 μg in PBS) were intravenously injected into C57BL/6 mice via the tail. After 12 h, the bilateral tibias and femurs were extracted to be detected by an IVIS spectrum imaging system (µCT45, Scanco Medical, Bassersdorf, Switzerland). The deposition of MVs in bones was evaluated by average radiant efficiency.

### Statistical analysis

The results are presented as the mean ± standard deviation (SD). All data were analyzed with GraphPad Prism 9 software. The statistical significance was analyzed by two-tailed Student’s *t* test or one-way analysis of variance (ANOVA) with Tukey’s post hoc tests. *P* < 0.05 was considered statistically significant.

### Supplementary information

Supplementary Information File

## Data Availability

All data generated in this study are available from the corresponding author upon request.
