# Supplementary material for: Annexin A5 derived from matrix vesicles protects against osteoporotic bone loss via mineralization
Source: Bone Res. 2023 Nov 9;11:60. doi: 10.1038/s41413-023-00290-9 (PMC10632518; doi:10.1038/s41413-023-00290-9)
Supplement: Supplementary file 1 — Supplementary Information File [file 41413_2023_290_MOESM1_ESM.doc]

Supplementary information for original article

**Annexin A5 derived from matrix vesicle protects against osteoporotic bone loss via mineralization**

Guanyue Su^1^, Demao Zhang^1^, Tiantian Li^1^, Tong Pei^1^, Jie Yang^1^, Shasha Tu^1^, Sijun Liu^1^, Jie Ren^1^, Yaojia Zhang^1^, Mengmeng Duan^2^, Xinrui Yang^1^, Yang Shen^1^, Chenchen Zhou^2^, Jing Xie^2^, Xiaoheng Liu^1^

^1^. Institute of Biomedical Engineering, West China School of Basic Medical Sciences & Forensic Medicine, Sichuan University, Chengdu 610041, China

^2^. State Key Laboratory of Oral Diseases, National Clinical Research Center for Oral Diseases, West China Hospital of Stomatology, Sichuan University, Chengdu 610041, China

**1. Supplementary figures**

**Figure S1**


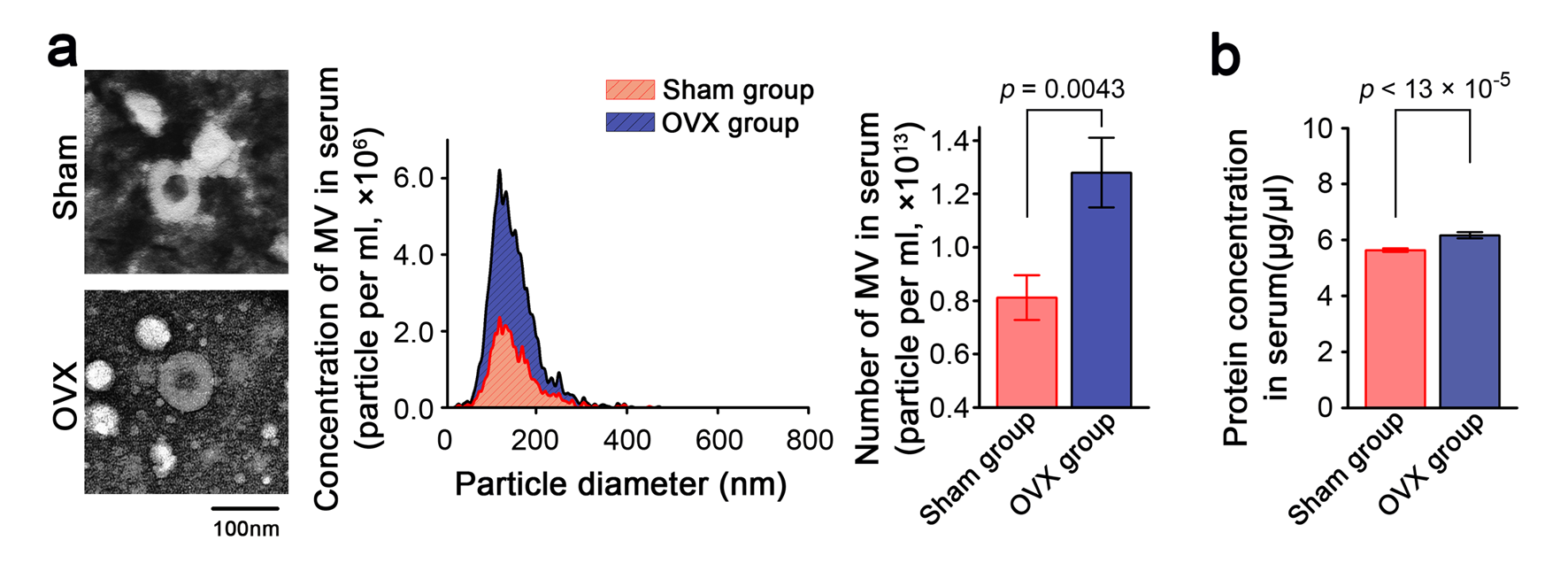


**Figure S1. Matrix vesicles isolated from serum increased in OVX mice.**

**(a)** TEM and NTA showing the changes of MVs isolated from the serum of normal and osteoporotic mice. TEM images (left) indicate the morphology of MVs isolated from both normal and osteoporotic serum; NTA (right) quantifies the changes in MV number between normal and osteoporotic serum. The data were based on five independent experiments (n = 5), and in an independent experiment, at least three mice were divided into the normal or osteoporotic group, respectively.

**(b)** BCA assay illustrating the changes of protein concentration of MVs isolated from both normal and osteoporotic serum. The data were based on five independent experiments (n = 5).

All data in **a** and **b** were presented as mean ± SD, and the significant data were based on two-tailed Student’s t-tests.

**Figure S2**

**
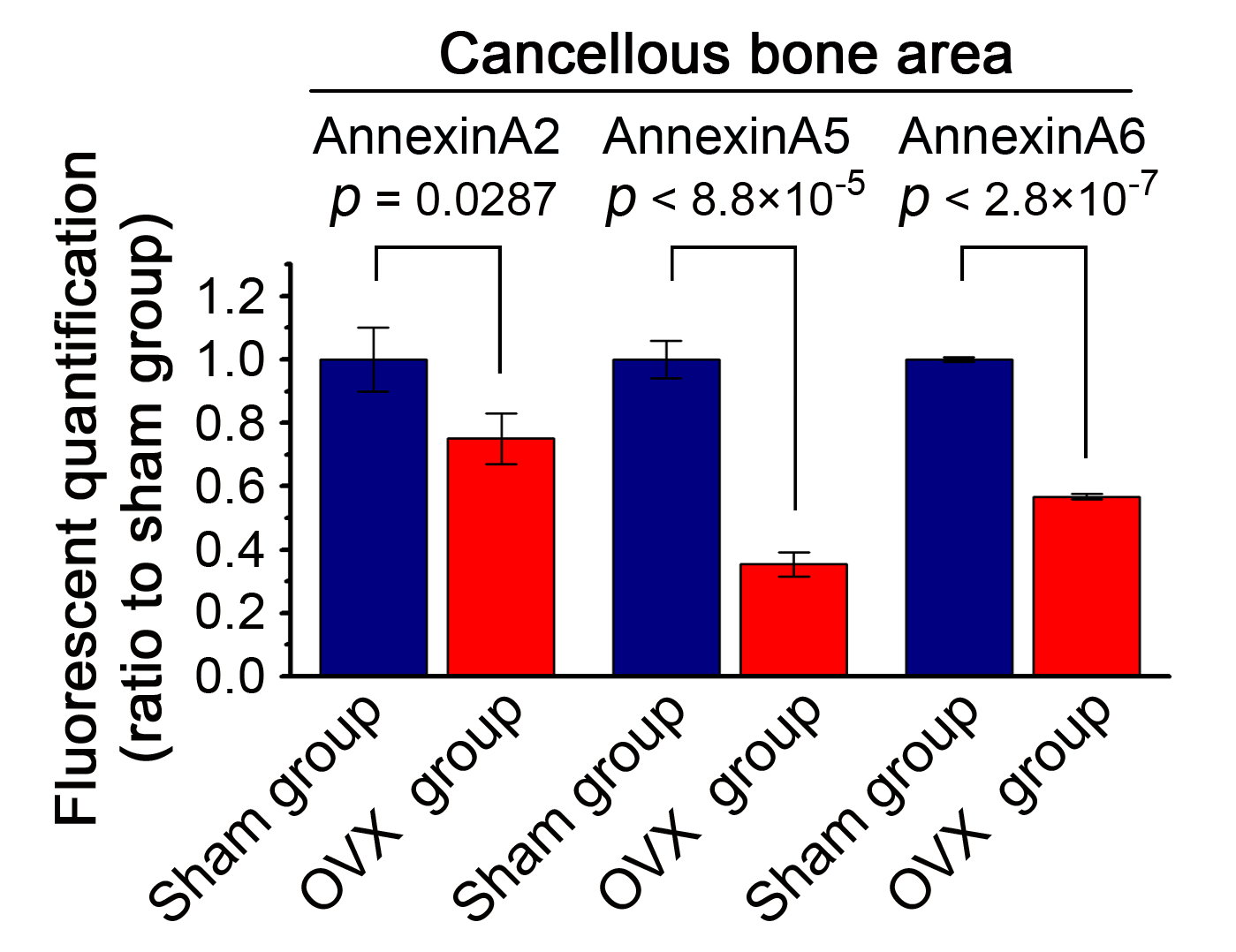
**

**Figure S2. Fluorescent OD quantification of protein changes of AnxA2, AnxA5 and AnxA6 in cancellous bone area.** The data were based on three independent experiments (n = 3) and presented as mean ± SD. The significant statistic analysis was based on two-tailed Student’s t-tests.

**Figure S3**

**
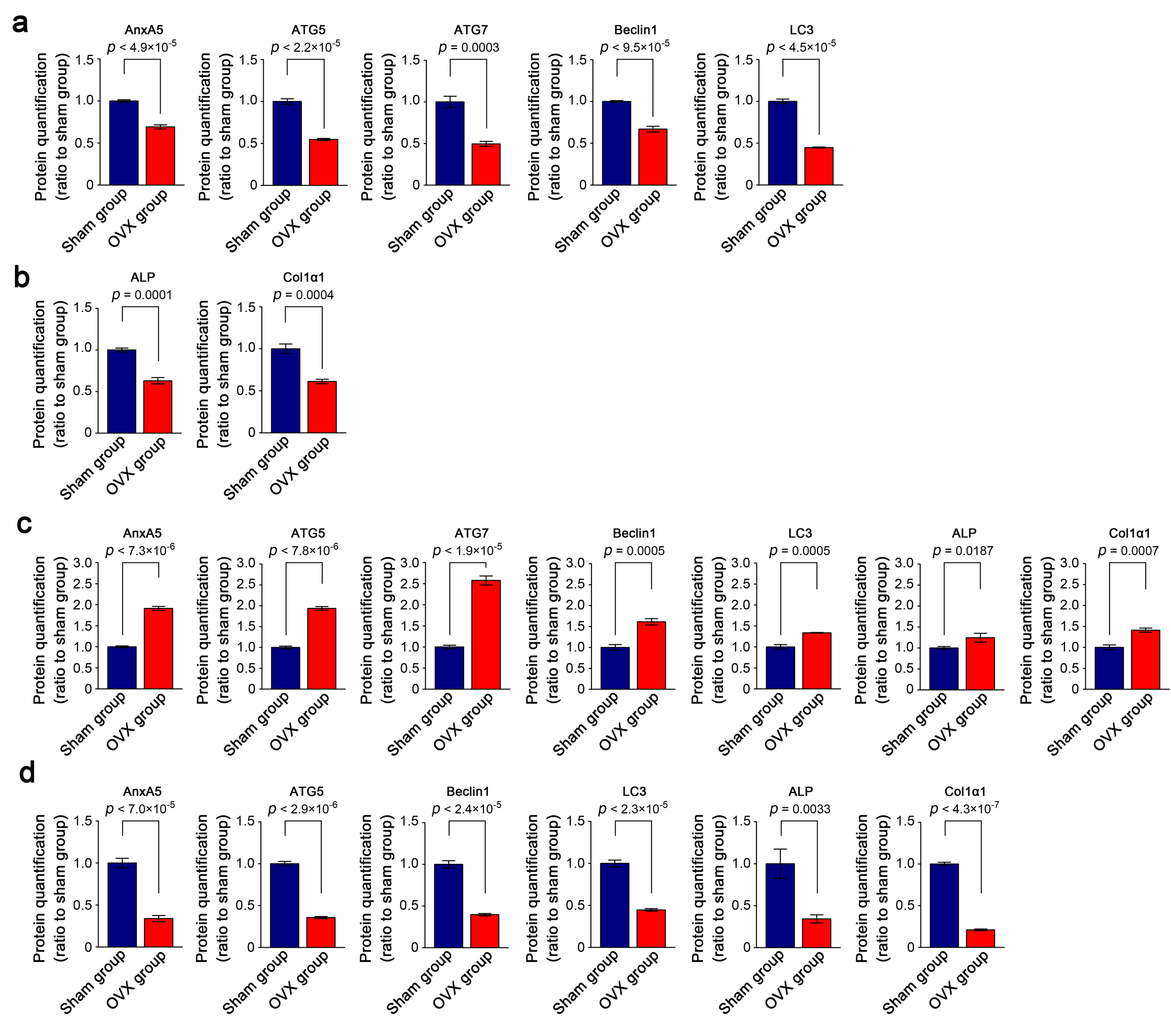
**

**Figure S3. Protein quantifications referred to figure 5.**

**(a)** Protein quantifications referred to figure 5b.

**(b)** Protein quantifications referred to figure 5c.

**(c)** Protein quantifications referred to figure 5d.

**(d)** Protein quantifications referred to figure 5e.

All data in **a** - **d** were based on three independent experiments (n = 3), and presented as mean ± SD. The significant statistical analysis was based on two-tailed Student’s t-tests.

**Figure S4**

**
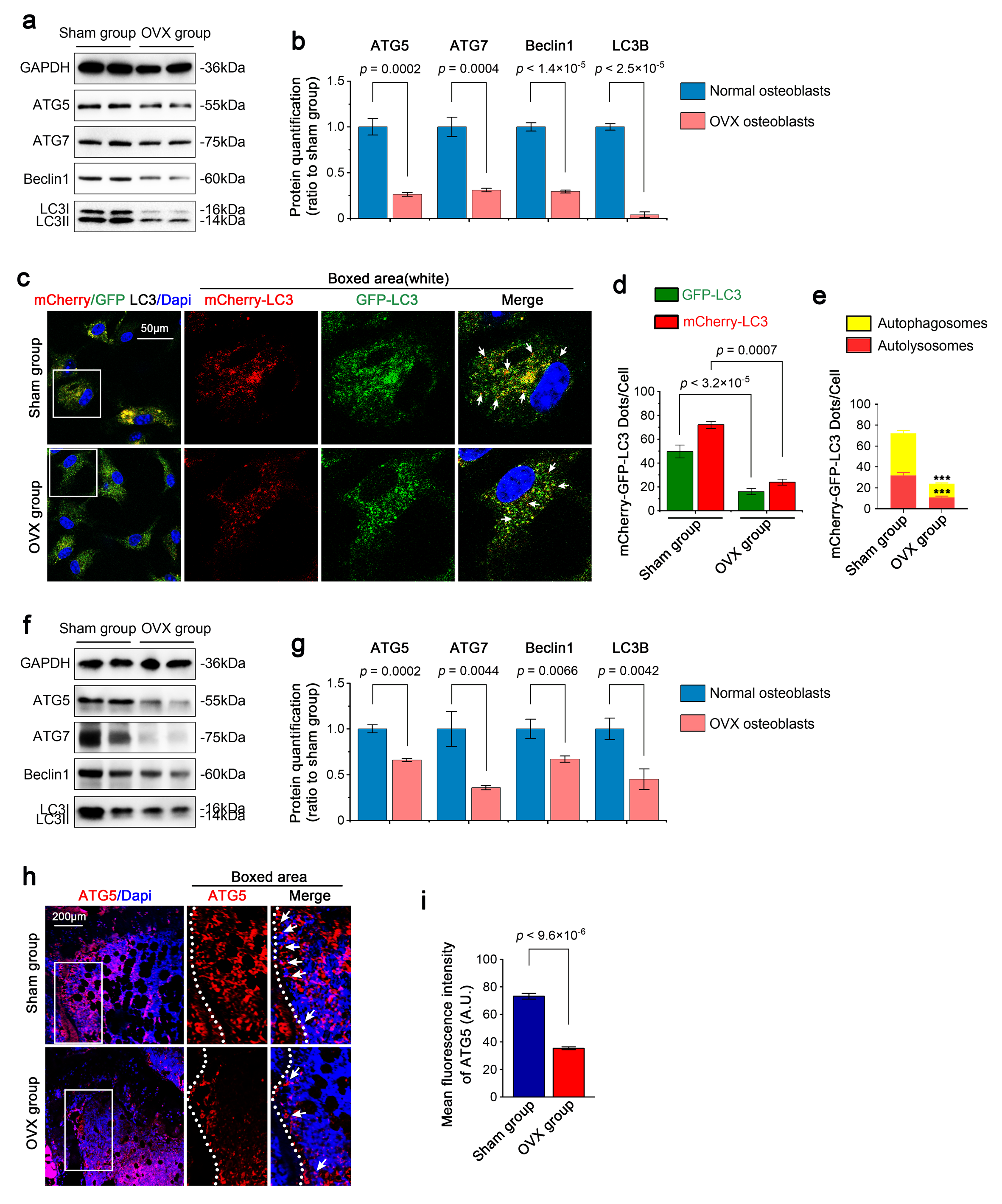
**

**Figure S4. Autophagy was impaired in the osteoblasts and bone tissue of OVX mice.**

**(a)** Western blotting showing the protein expressions of autophagic-related markers ATG5, ATG7, Beclin1 and LC3B in primary osteoblasts from normal and osteoporotic bone. The images were based on three independent experiments (n = 3).

**(b)** OD quantification confirming the protein changes of autophagic-related markers ATG5, ATG7, Beclin1 and LC3B in primary osteoblasts from normal and osteoporotic bone. The data were based on three independent experiments (n = 3).

**(c)** Ad-mCherry-GFP-LC3 transfection assay showing the occurrence of autophagy in primary osteoblasts from normal and osteoporotic bone. The yellow mCherry-GFP-LC3 dots were indicated by white arrows, which indicate the formation of autophagosomes. The images were chosen based on three independent experiments (n = 3).

**(d)** Quantitative analysis of the number of mCherry and GFP dots in primary osteoblasts from normal and osteoporotic bone shown in (c).

**(e)** Quantitative analysis of the number of autophagosomes and autolysosomes in primary osteoblasts from normal and osteoporotic bone shown in (c).

**(f)** Western blotting showing the protein expressions of autophagic-related markers ATG5, ATG7, Beclin1 and LC3B in long bones of normal and osteoporotic mice. The images were chosen based on three independent experiments (n = 3).

**(g)** OD quantification confirming the protein changes of autophagic-related markers ATG5, ATG7, Beclin1 and LC3B in long bones of normal and osteoporotic mice. The data were based on three independent experiments (n = 3).

**(h)** Immunofluorescence by CLSM showing the distribution and expressions of ATG5 in normal and osteoporotic cancellous bones (red: ATG5, blue: nucleus). White dashed lines indicate the expression of ATG5 at the sites of osteoblasts distribution. Arrows further show the details of ATG5 at the site of osteoblasts. The images were chosen based on three independent experiments (n = 3).

**(i)** Immunofluorescent OD quantification confirming the protein changes of autophagic-related markers ATG5 in normal and osteoporotic cancellous bones (red: ATG5, blue: nucleus).

All data in **b**, **d, e, g** and **i** were presented as mean ± SD, and the significant data were based on two-tailed Student’s t-tests.

**Figure S5**


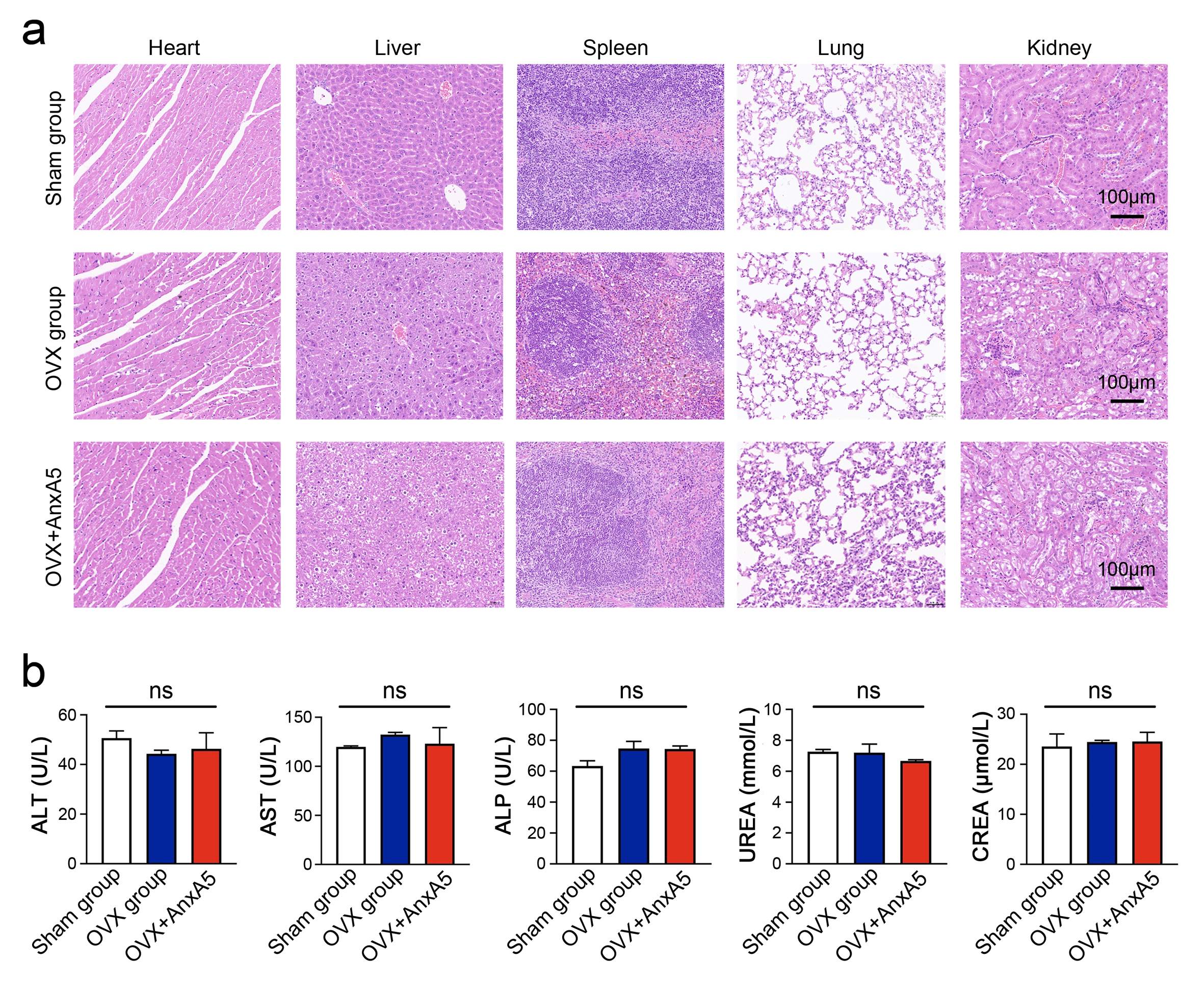


**Figure S5. Systemic toxicity evaluation after tail intravenous injection.**

**(a)** Representative H&E-staining images showing the morphological changes of main organs in normal, OVX and OVX+AnxA5 mice after 12 h injection. The images were chosen based on three independent experiments (n = 3).

**(b)** Biochemical assay detecting the changes of ALT, AST, ALP, UREA and CREA in serum after AnxA5 administration for 2 month (AnxA5 injection twice a week). The experiment was based on three independent experiment (n=3) and presented as mean ± SD. The significant data were based on one way analysis of variance (ANOVA) with Tukey’s post hoc tests. ALT: Alanine transaminase; AST: Aspartate aminotransferase; ALP: Alkaline phosphatase; UREA: Urea; CREA: Creatinine.

**2. Supplementary tables**

Table S1 Detailed information of antibodies

| **Category** | **Antibody** | **Isotype** | **Manufacturer** | **Cat. No** | **Dilution** |
| --- | --- | --- | --- | --- | --- |
| **Annexin** | AnxA2 | Mouse | Proteintech | 60051-1-lg | 1:1000 |
|  | AnxA5 | Rabbit | abcam | ab14196 | 1:1000 |
|  | AnxA6 | Rabbit | Proteintech | 12542-1-AP | 1:500 |
| **Osteoblastic differentiation markers** | ALP | Rabbit | HuaBio | ET1601-21 | 1:1000 |
|  | Col1α1 | Rabbit | Proteintech | 14695-1-AP | 1:1000 |
| **Autophagy markers** | Beclin1 | Rabbit | Santa Cruz | sc‐11427 | 1:100 |
|  | ATG5 | Rabbit | HuaBio | ET1611-38 | 1:1000 |
|  | ATG7 | Mouse | Proteintech | 67341-1-Ig | 1:1000 |
|  | LC3 | Rabbit | Abcam | ab192890 | 1:1000 |
| **Internal control** | GAPDH | Rabbit | SAB | 21337 | 1:1000 |
